# The lung in acute respiratory failure: insights from synchrotron radiation imaging

**DOI:** 10.1186/s40635-026-00896-3

**Published:** 2026-05-08

**Authors:** Sam Bayat, Gergely Albu, Luca Fardin, Alberto Bravin

**Affiliations:** 1 Inserm UA07 STROBE Laboratory, Université Grenoble Alpes, Grenoble, France; 2 Department of Pulmonology and Physiology, Grenoble University Hospital, Bd. Du Maquis du Grésivaudan, La Tronche, 38700 Grenoble, France; 3 Division of Anaesthesiology, Department of Acute Care Medicine, Geneva University Hospitals, Geneva, Switzerland; 4 Elettra Sincrotrone, Trieste, Italy; 5 Dipartimento di Fisica, Università degli Studi di Milano-Bicocca, Milan, Italy; 6 Dipartimento di Fisica, Università della Calabria, Arcavacata di Rende, Italy; 7 Istituto Nazionale di Fisica Nucleare, Sezione di Milano-Bicocca, Milan, Italy; 8 Sezione di Rende, CNR-Nanotec, Lecce, Italy

**Keywords:** Synchrotron radiation, Phase-contrast CT, K-edge subtraction, ARDS, Ventilator-induced lung injury, Airway closure, Extracellular matrix, 4D imaging, Regional lung ventilation, Regional lung perfusion, Atelectrauma

## Abstract

Acute respiratory failure and the acute respiratory distress syndrome (ARDS) are characterized by profound spatial and temporal heterogeneity in lung aeration, mechanics, and inflammation. While mechanical ventilation is often vital to ensure gas exchange, it can also cause or worsen lung injury as a result of the superimposed mechanical stress due to the application of positive pressure on a heterogeneous parenchyma. Characterizing the lung microstructure and function at small length scales is essential for understanding the pathogenesis of ventilator-induced lung injury. This is a challenging task due to the complex architecture of the lung, to its constant motion and deformation with breathing and pulsatile blood flow, and its multiscale organization. Indeed, mechanical forces act on the components of the extracellular matrix and cells, acini, airways and blood vessels at the microscale to impact numerous biological processes. Beyond these effects, the global mechanical behavior and function of the lung emerge from this complex dynamic system. Because synchrotron radiation imaging techniques such as phase-contrast computed tomography (PC-CT), and K-edge subtraction CT (KES-CT), have a high spatial resolution, are quantitative, and are fast, they offer unique insights into the multiscale, dynamic behavior of the lung. These modalities enable mapping of regional ventilation, blood distribution, within-breath alveolar recruitment/derecruitment, airway closure, and tissue strain. This review explains synchrotron radiation imaging modalities, and discusses the findings in models of acute respiratory failure, their translational implications, as well as future areas of investigation.

## Introduction

Acute respiratory failure (ARF) is a common reason for admission to the intensive care unit. Acute respiratory distress syndrome (ARDS), a severe manifestation of ARF, arises from widespread lung inflammation, parenchymal heterogeneity, and high microvascular permeability edema [1], resulting in lung volume loss and hypoxemia. Mechanical ventilation is a critical treatment for severe respiratory failure, yet it can exacerbate lung injury by exposing mechanically inhomogeneous lung tissues to excessive stress and strain, a phenomenon known as ventilator-induced lung injury (VILI). The detailed mechanism of VILI at the microscopic level remains unclear. Factors such as excessive lung tissue stretch through hyperinflation [2], cyclic alveolar recruitment and derecruitment or “atelectrauma” [3], increased energy dissipation in the parenchyma [4], and related inflammation [5] can all contribute to the worsening of lung injury [6]. The advent of protective mechanical ventilation has significantly decreased mortality rates [7]. Current treatment strategies focus on reducing tidal volume and preventing atelectrauma by applying positive end-expiratory pressure (PEEP). Despite these interventions, ARDS mortality rates remain high [7]. In the later stages of ARDS, the repair of alveolar epithelium involves interactions among various alveolar cell types and the extracellular matrix (ECM). During this phase, fibroproliferation induced by immune cells and inflammatory mediators can lead to fibrosis due to excessive collagen deposition in surviving ARDS patients [6].

Clinical CT imaging is crucial for diagnosing ARDS, providing both morphological [8], and more recently functional [9, 10], characterization of the lungs. However, clinical CT lacks the spatial and temporal resolution necessary to image phenomena occurring at the scale of pulmonary acini and alveoli. Synchrotron radiation (SR) microtomography addresses some of these limitations [11]. The high flux and unique properties of synchrotron X-ray beams enable functional and structural imaging at spatial resolutions as fine as a few micrometers, with temporal resolutions sufficient to capture cardiac and ventilatory motion.

SR-based techniques have been utilized in lung imaging. Among these techniques, K-edge subtraction CT (KES-CT) leverages the high flux and energy tunability of SR beams to map the distribution of contrast elements [12]. Phase-contrast synchrotron radiation CT (PC-CT) utilizes a property of SR beams known as coherence, which allows for the use of X-ray refraction and scattering as an additional contrast source when imaging small, poorly attenuating tissues that are otherwise invisible with conventional CT [13]. Four-dimensional (4D) implementations of these techniques provide detailed maps of local tissue strain and its temporal evolution during mechanical ventilation [13]. Moreover, X-ray diffraction at the nanoscale reveals the periodic organization of hierarchical molecular components of the extracellular matrix (ECM), which can be mapped ex vivo in macroscopic tissue samples [14].

This review aims to synthesize key insights provided by SR imaging in models of acute respiratory failure and to discuss their implications for ventilator-induced lung injury and the development of personalized ventilation strategies. We begin by briefly outlining the main synchrotron imaging modalities and then review findings on regional ventilation and aeration, airway closure and atelectrauma, ECM and nanoscale remodeling, before discussing translational perspectives.

### Synchrotron radiation imaging modalities

SR facilities are electron accelerators that generate electromagnetic radiation when high-energy electrons travel through the magnetic fields of bending magnets and insertion devices. The radiation that is produced, or synchrotron radiation, is characterized by an extremely high photon flux, over an energy range that can span from infrared to high-energy X-rays. The high flux, coupled to dedicated optical elements, allows for a precise selection of the photon energy, yielding high-intensity monochromatic beams. Additionally, the radiation is highly collimated and can be propagated over long distances. This property, coupled to a micrometric source size, produces a high degree of spatial coherence, an essential property for advanced X-ray imaging techniques.

**Fig. 1:**
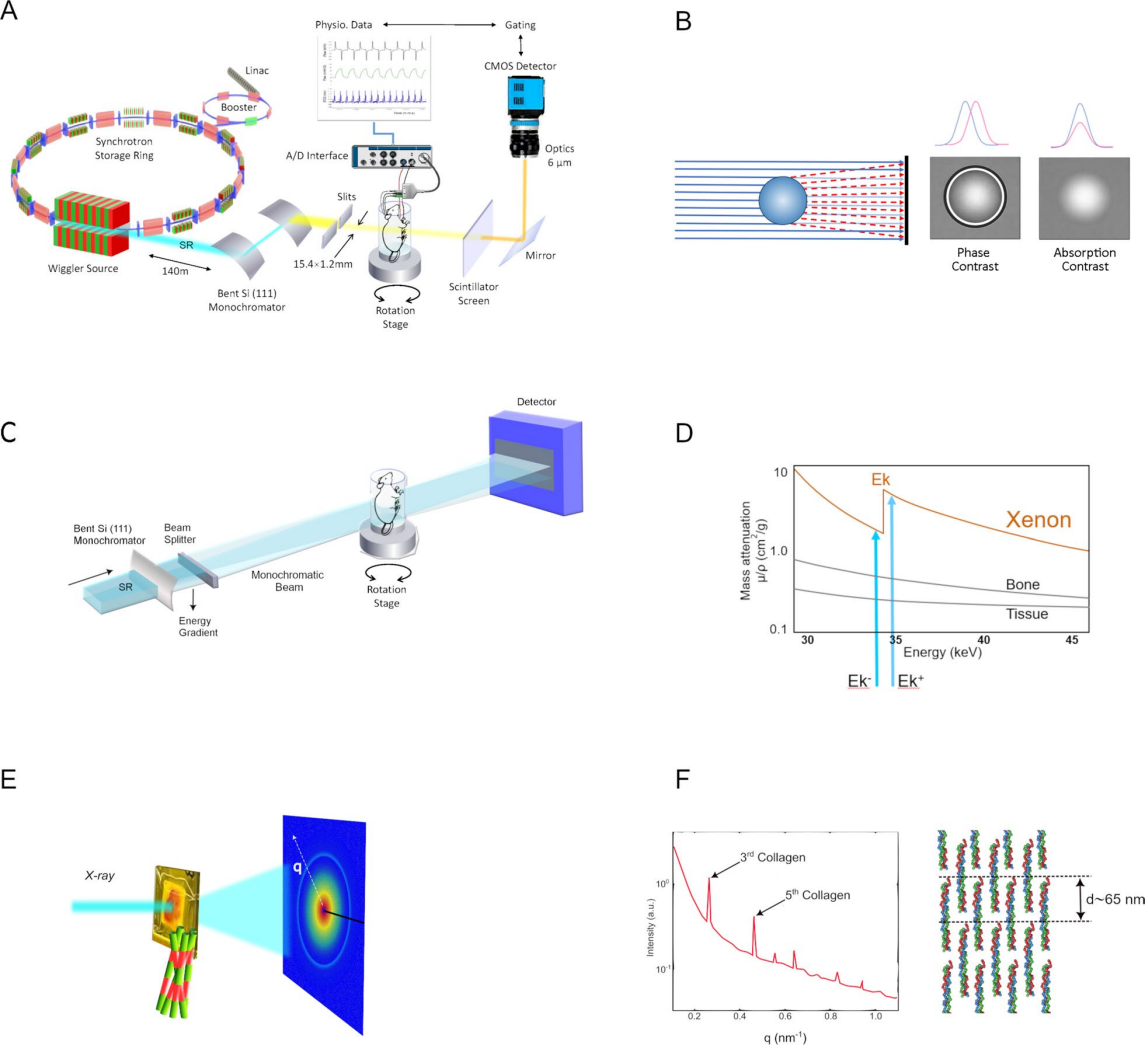
Schematic overview of synchrotron-radiation (SR) imaging modalities. **A** 4D X-ray phase-contrast microtomography of rat lung using a synchrotron X-ray source. High-intensity coherent X-rays generated from electrons orbiting in a storage ring, are rendered monochromatic using bent silicon crystal optics, and detected by a fast camera coupled to a scintillator and optics yielding an isotropic pixel size of 6 µm^3^. The animal is mechanically ventilated while the electrocardiogram and respiration are monitored and used for cardiorespiratory image gating. **B** Phase and absorption contrast. X-rays crossing the sample are absorbed as in classical radiography, but also refracted, which causes phase changes. Interaction of refracted and transmitted X-rays is a source of contrast due to small differences in refractive index within the sample. **C** Set-up for K-edge imaging. Two monochromatic beams are generated from the full radiation spectrum with a bent silicon crystal. The degree of bending changes the vertical energy gradient in the beam. The 2 beams are focused on the sample, and have energies that bracket the K-edge of the contrast agent. **D** CT images were acquired simultaneously above (Ek +) and below (Ek-) the K-edge of Xe, exploiting the abrupt change in X-ray attenuation of Xe to compute quantitative distribution maps. **E** SAXS. The lung sample is irradiated by monochromatic X-ray generated by a synchrotron. The ordered fibrillar organization of collagen in the sample produces an X-ray scattering pattern recorded on the detector. **F** Azimuthal integration of the diffraction pattern produces an intensity vs diffraction angle (q) curve, where the peaks are due to the d-periodicity of the collagen fibrils. The D-spacing of collagen fibrils is related to q by q = 2π/d

These properties underpin two key advantages for lung imaging. First, coherence enables phase-contrast imaging [13, 15] (Fig. 1A), which is highly sensitive to differences in refractive index at the numerous air–tissue interfaces within the lung, and greatly enhances soft-tissue visibility and contrast compared with purely absorption-based imaging [16] (Fig. 1B).

Second, the high flux of SR and the possibility to precisely select the energy of the derived monochromatic beams enable K-edge subtraction (KES) imaging [17] (Fig. 1C). KES-CT exploits the abrupt change in X-ray attenuation at a contrast agent’s K-edge to simultaneously measure parenchymal density and contrast concentration (Fig. 1D). Two or more CT data sets are acquired for this purpose at monochromatic energies slightly below and above the contrast element’s K-edge. Material decomposition algorithms combine these datasets to yield quantitative maps of a contrast element’s concentration and, simultaneously, of tissue density [18]. In lung applications, stable xenon is used as an inhaled high-atomic-number contrast gas [19], and iodine or gadolinium can be used as an intravascular contrast element [20].

An alternative strategy relies on dual-energy computed tomography (DECT) implemented with conventional clinical X-ray systems [21, 22]. Unlike synchrotron sources that provide monochromatic radiation, clinical CT scanners generate polychromatic X-ray spectra [23]. Nevertheless, the conceptual basis underlying K-edge-based material discrimination can still be applied. Computational methods have been developed to estimate the composition of scanned tissues and to separate contrast agents from native tissue components. To enable dual-energy acquisition in clinical scanners, several technical solutions have been introduced, including dual X-ray sources and photon-counting detectors [22]. These approaches have enabled functional imaging of regional pulmonary ventilation, for example through the use of inhaled xenon gas experimentally [24], or in patients with conditions such as COPD, asthma, and bronchiolitis obliterans [25]. Also, the perfused blood volume can be mapped as a surrogate of parenchymal perfusion [26, 27]. Despite these advances, the clinical application of DECT for this purpose remains constrained by several technical limitations. These include radiation exposure, partial overlap between the energy spectra used for material separation, image noise, beam-hardening artifacts, and the need for meticulous calibration of the imaging system [22].

**Fig. 2:**
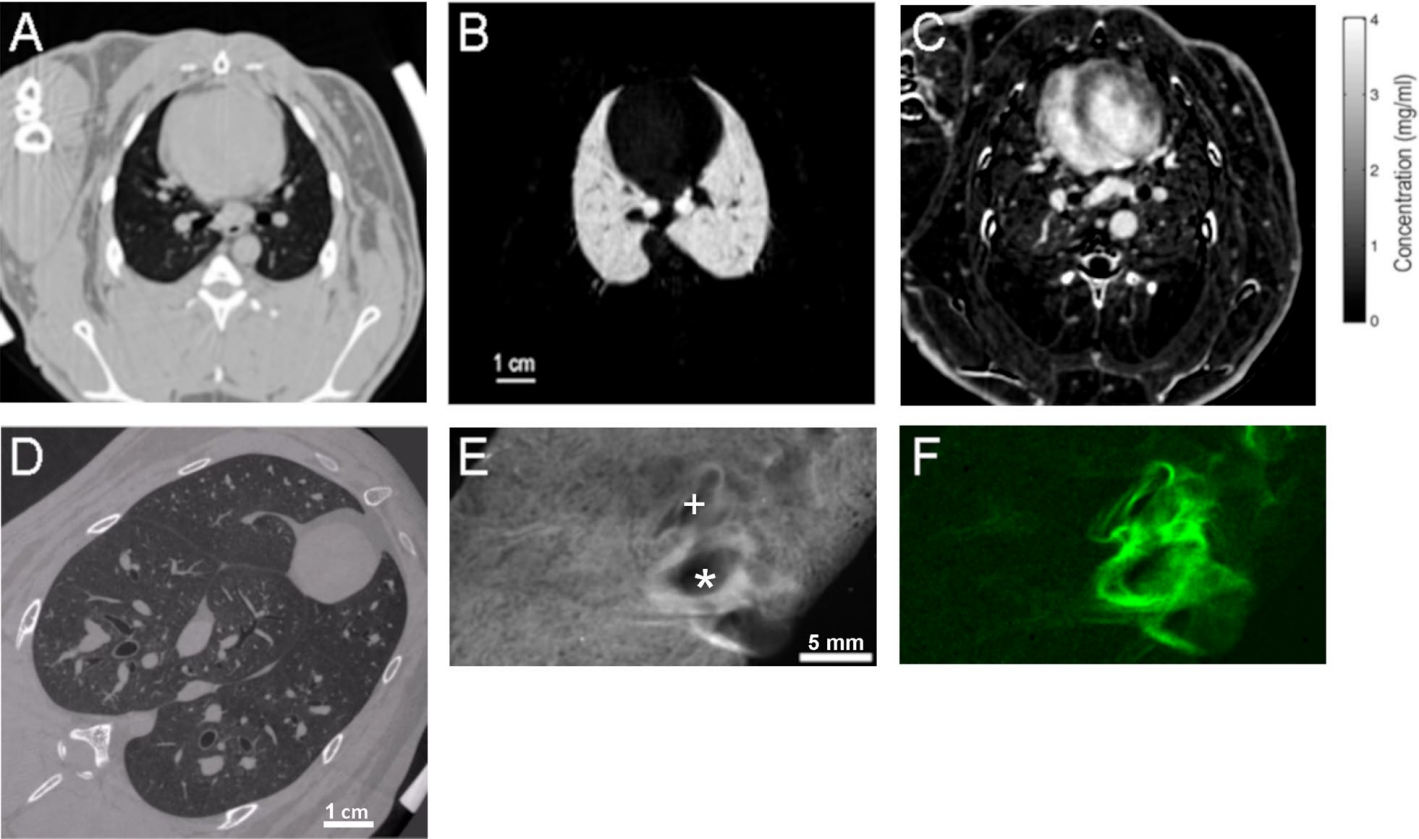
Sample K-edge subtraction CT images in a normal rabbit lung acquired in vivo at 350 µm voxel resolution; **A** tissue density; **B** xenon concentration; **C** iodine concentration distributions, B & C are quantitative maps coded in grey scale (grayscale bar). A, B and C were acquired in the same animal. **D** A sample synchrotron phase-contrast CT image in a different animal at 20 µm voxel resolution, acquired in vivo with respiratory and cardiac gating. **E** total scattering signal intensity; and F corresponding background-corrected intensity of 3rd-order collagen peak (green) in a snap-frozen post-mortem normal lung sample. Note the stronger collagen scattering signal around a bronchus (*) and a neighboring blood vessel ( +)

**Fig. 3:**
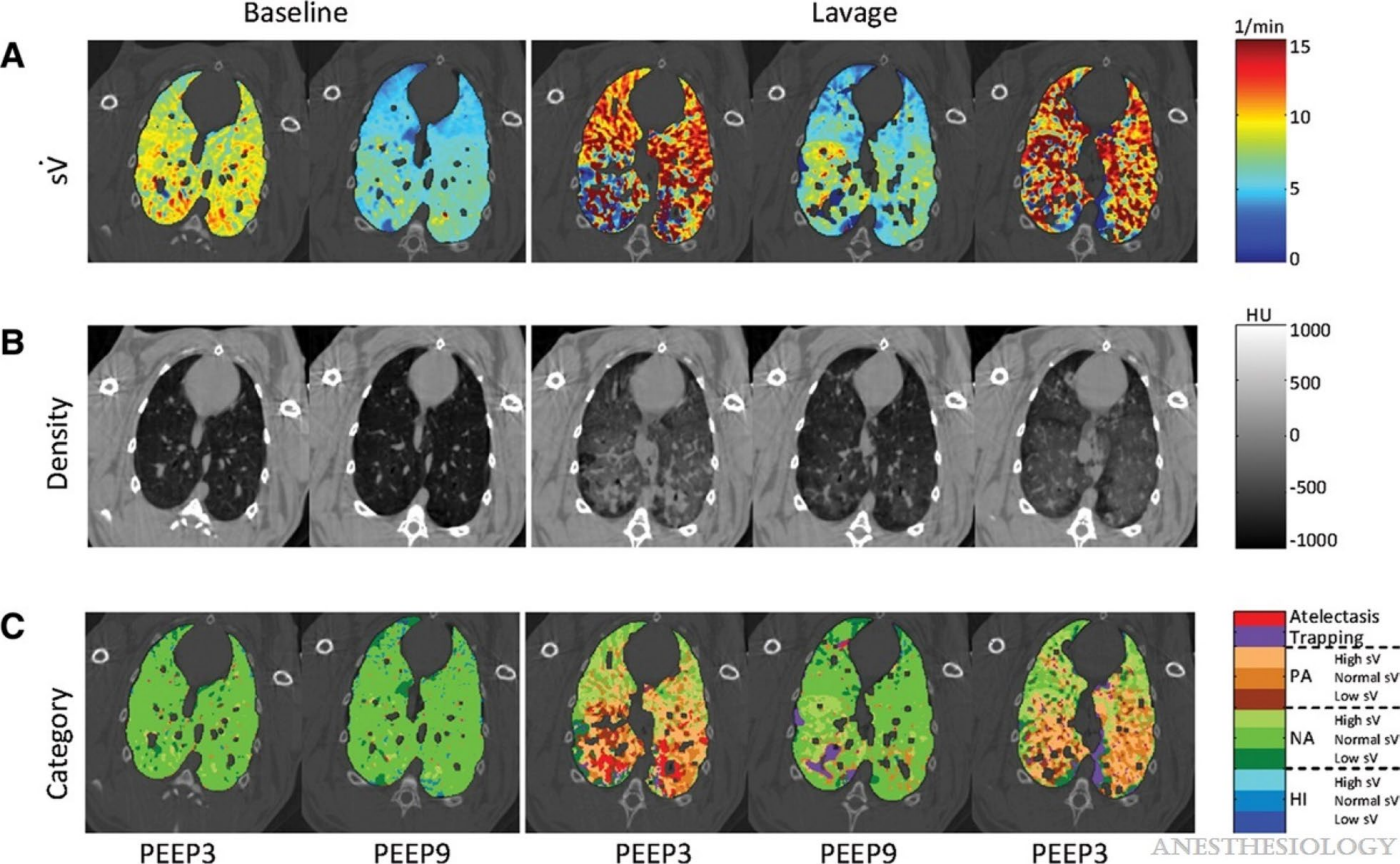
Sample composite images showing the distribution of specific ventilation maps ($s\dot{V}$) obtained with synchrotron KES imaging (**A**), and tissue density (**B**), in a dependent image slice in a representative rabbit. **C** Spatial distribution of lung regions defined as atelectatic, trapped, poorly aerated (PA) normally aerated (NA) or hyperinflated (HI) based on aeration. Color scale indicates subregions with high, normal, or low $s\dot{V}$ within PA, NA, and HI categories. Reproduced with permission from [32]

Small-angle X-ray scattering (SAXS) is based on the elastic scattering of photons by macromolecules: when a monochromatic wave hits an object, the electrons of its atoms become sources of secondary waves (Fig. 1E). Despite the random orientation of macromolecules in biological samples, the scattering pattern contains peaks arising from the periodic organization of some biomolecules at the nanoscale [28] (Fig. 1F). SAXS is nondestructive, and provides maps of structural data over macroscopic sample volumes. It has the advantage of scanning much larger samples than transmission electron microscopy [14]. Examples of synchrotron KES, phase-contrast CT images, and SAXS maps are shown in Fig. 2. Sample in vivo 4D phase-contrast lung images at 6 µm voxel resolution are shown in Fig. 3A.

Synchrotron-based X-ray tomographic microscopy (SRXTM) has been used to reconstruct real three-dimensional alveolar geometries with sub-micron resolution [11]. SRXTM uses the high flux and monochromaticity of X-rays produced with a synchrotron to acquire high-resolution (< 1 μm^3^ voxel resolution) and higher-contrast volumetric images in much shorter acquisition times than laboratory micro-CT. Ex vivo SRXTM combined with image processing allows the rapid quantitative assessment of 3D microstructures, enabling virtual histology [29].

### Regional lung structure and function in preclinical models of ARDS

KES-CT studies in preclinical models of ARF consistently show that the mechanical behavior of the peripheral lung is profoundly altered, in a way that is difficult to appreciate by global measurements at the airway opening. Peripheral lung units can be conceptualized as compliant airspaces subtended by the terminal branches of the airway tree. The local distribution of ventilation is governed by both the resistance of airways and the compliance of respiratory tissues, the product of which describes a time constant [30]. Units with higher airway resistance or tissue compliance will have a reduced rate of gas renewal (increased time constant). Moreover, lung tissue is viscoelastic: like many biological tissues, the response of the lung parenchyma to stress is delayed, as it is impeded by internal frictional forces [31].

**Fig. 4:**
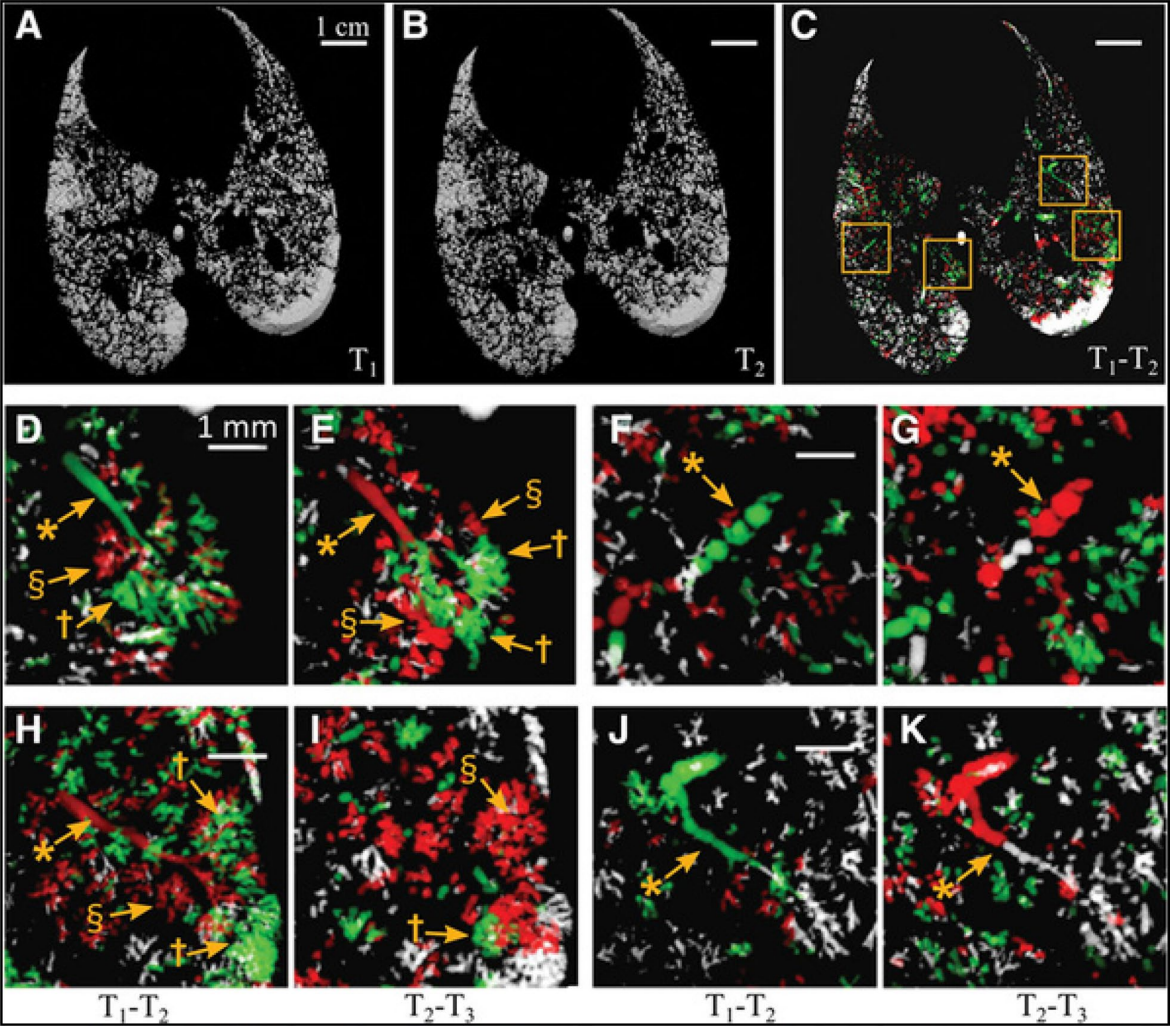
In vivo recruitment/derecruitment of airspaces characterized by phase-contrast CT. **A–E** Alveolar recruitment/derecruitment (R/D) occurs alternately in neighboring lung units over short time scales ~ 1 min, in injured lung at positive end-expiratory pressure (PEEP) 6 cm H2O. **A–C** 3D renderings of aerated lung regions obtained by segmentation of synchrotron phase-contrast CT images at 0 (**A**) and 84 s (**B**) in a 2.5 mm thick slice, in injured lung at PEEP 6 cm H_2_O;** C**, R/D map is quantified using image registration between T1 (**A**) and T2 (**B**). White: aerated, no change; black: nonaerated, no change; green: opening; and red: closing. Yellow squares delineate regions of interest magnified in **D–E** with **D**, **F**, **H**, and** J** computed from two successive images acquired at 0 and 84 s (T1–T2) and **E**, **G**, **I**, and** K** from the subsequent time interval between 84 and 159 s (T2–T3). *Bronchioles, †recruiting airspaces, and §derecruiting airspaces. Reproduced with permission from [41]

KES-CT simultaneously mapping aeration and ventilation in a whole-lung lavage model of ARDS revealed a continuum of lung functional states, from atelectasis and poor aeration to normal aeration, gas trapping, and hyperinflation (Fig. 4) [32]. Poorly aerated dorsal regions remained ventilated, consistent with tidal recruitment–derecruitment (R/D) and increased ventilation inhomogeneity. Expansion of aerated alveoli adjacent to collapsed regions created a heterogeneous stress distribution, while ventilation was redistributed toward normally aerated ventral regions, increasing the risk of overstretch and ventilator-induced lung injury [33–35]. Moderate PEEP reduced atelectasis but promoted hyperinflation of normally aerated regions. Conversely, pressure reduction increased poor aeration and gas trapping, reflecting instability and expiratory closure of small airways in injured lungs [35]. Exogenous surfactant recruited atelectatic regions, converting them into poorly aerated but ventilated units and improving global respiratory mechanics and gas exchange [35].

Deflation studies with KES-CT have provided complementary insight into alveolar derecruitment mechanisms. Scaramuzzo et al. analyzed how the number and surface area of airspaces change during stepwise PEEP reduction in healthy lungs [36]. They showed that loss of lung volume with decreasing PEEP was driven mainly by a reduction in airspace numerosity rather than a uniform reduction in size, consistent with discrete derecruitment events. In a subsequent ARDS model, the same group demonstrated that in injured lungs, both airspace size and number decreased with PEEP reduction, and that deflation behavior differed between concentric regions of interest, reflecting heterogeneous micromechanics and strain gradients [37].

Similar patterns of spatial heterogeneity have been reported with other functional imaging modalities. Hyperpolarized gas MRI studies have shown that loss of aeration in injured lungs can amplify stretch in adjacent aerated regions and promote propagation of lung injury, findings consistent with the redistribution of ventilation and stress observed with SR imaging [38]. Likewise, positron emission tomography using intravenously infused ^13^N tracers has demonstrated marked ventilation–perfusion heterogeneity during bronchoconstriction and acute lung injury, as well as redistribution of perfusion with changes in airway pressure, supporting the concept that regional mechanical heterogeneity strongly shapes gas exchange in injured lungs [39, 40]. What is unique to synchrotron KES imaging is the possibility to depict both tissue aeration and ventilation or perfused blood volume using a single imaging modality, and at higher spatial and temporal resolutions than other functional imaging techniques [13].

#### Dynamics of atelectrauma

**Fig. 5:**
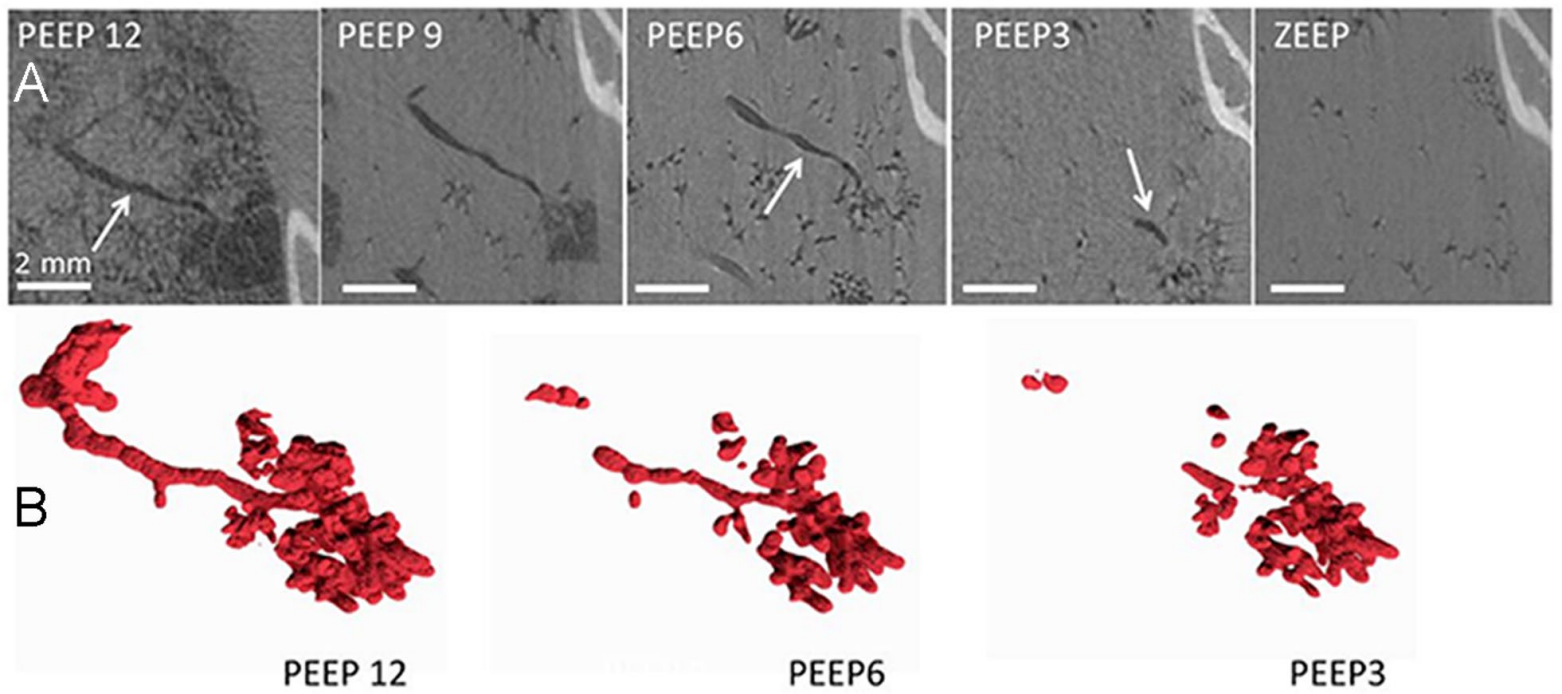
**A** In vivo airway closure characterized by phase-contrast CT. **B** 3D reconstruction of the compliant closure of a terminal airway resulting in distal air trapping in the subtended acinus, as airway pressure is decreased stepwise from 12 cmH_2_O positive end-expiratory pressure (PEEP) to 0 cmH_2_O (ZEEP). Reproduced with permission from [51]

Broche et al. used SR PC-CT to investigate cyclic alveolar R/D during mechanical ventilation in a lavage-induced ARDS model in anaesthetized rabbits [41]. They sequentially acquired PC-CT images at subacinar resolution over short intervals of _~_1.5 min, with identical pressure conditions. They repeated imaging at end-expiratory pressures ranging from 0 to 12 cmH₂O. By registering and subtracting consecutive 3D volumes (Fig. 5, A–C), the authors mapped the extent and spatial distribution of R/D with high sensitivity. They observed alternating patterns of opening and closing within the same time frame in neighboring and even communicating airspaces subtended by the same terminal airway, presumably exposed to the same pressure, demonstrating that R/D is both highly heterogeneous and temporally asynchronous at the subacinar scale. These findings were novel compared with the inferences from the intravital microscopy studies that were limited to subpleural airspaces [42].

To gain deeper insights from their observations, the authors integrated their imaging data with computational modeling based on a realistic rabbit lung geometry, reconstructed from a segmented bronchial tree. In this model, the opening and closing of individual airways were controlled not only by critical opening pressures but also occurred at a defined, finite speed rather than instantaneously [43]. In other words, alveoli that can be recruited will open once a critical opening pressure is surpassed, but this occurs with a delay, which is influenced by tissue micromechanical properties and airway liquid surface tension. Nonetheless, this characteristic alone was insufficient to replicate the asynchronous, sometimes opposing behavior of neighboring acini observed in vivo. In contrast, when mechanical tethering between these acinar structures was combined with the time-dependent nature of recruitment and derecruitment (R/D), the model successfully mirrored the in vivo findings. This implies that mechanical interdependence dynamically alters the critical pressures for airway opening and closing.

Fardin et al. expanded these studies by using 4D phase-contrast computed tomography (PC-CT) to image the within-tidal dynamics of cyclic R/D, and examining the relationship between R/D and parenchymal cellular infiltration as an indicator of inflammation, in an acute respiratory distress syndrome (ARDS) model in rabbits [44]. This technique enabled them to capture videos and quantitative maps of within-tidal R/D under protective mechanical ventilation. They confirmed that R/D of peripheral airspaces was dependent on both pressure and time, occurring throughout the respiratory cycle with significant variability in opening and closing pressures. Additionally, the progression of lung injury under positive pressure ventilation extended outward from nonaerated regions to adjacent areas where cyclic R/D was evident. This finding was in line with conventional CT imaging studies in rat lung [45]. Moreover, there was a notable correlation between R/D and regional lung cellular infiltration, indicating that tidal R/D of the lung parenchyma may contribute to regional lung inflammation and capillary–alveolar barrier dysfunction, as well as the progression of lung injury. This finding was new in comparison to other imaging studies of lung injury. Positive end-expiratory pressure (PEEP) did not fully alleviate this phenomenon, even at high levels. A crucial finding of their research was the coexistence of recruitment and derecruitment throughout the respiratory cycle in ARDS. Although seemingly counterintuitive, one explanation for this finding is the pronounced time-dependence of cyclic R/D, attributed to the viscoelastic nature of alveolar opening and collapse. Moreover, the recruitment pattern is not continuous but rather occurs as a series of “avalanches” [46], causing small amplitude oscillations in respiratory flow.

An even more precise link between local parenchymal stretch and the expression of inflammatory mediator gene expression was shown by Yen et al. [47]. They imaged groups of BALB/c mice under protective and injurious ventilation using 4D PC-CT, not using a synchrotron X-ray source per se, but a liquid MetalJet X-Ray Source Technology (Excillum AB, Sweden), enabling high-brightness and high-resolution imaging. They found statistically significant associations between regional tidal stretch and Ccl2 and IL-6 expression. The Ccl2 but not IL-6 protein expression replicated the gene data. The increased expression of genes involved in cell motility/chemotaxis, cell adhesion, and inflammation as a result of increased tidal strain under mechanical ventilation has been reported in other studies in rodents, and in pigs imaged with positron emission tomography [48, 49].

#### Airway closure

For a long time, clinicians and respiratory physiologists had suspected that small airways closure and atelectasis play a prominent role in ARDS [50]. This hypothesis was based on a large amount of indirect evidence; however, direct in vivo demonstration of small airways closure in ARDS was lacking. Broche et al. used Phase-contrast CT to characterize small-airway behavior over a range of PEEP levels in a preclinical ARDS model induced by surfactant depletion followed by injurious mechanical ventilation [51]. Cross-sectional areas of the same individual airways were measured at multiple pressures (Fig. 5). Compared with baseline, injured lungs exhibited increased airway collapsibility, manifested by a steeper decline in airway caliber as pressure was reduced. This effect was most pronounced in small airways with radii between _~_200 µm and 1.6 mm at 12 cmH₂O.

These experiments identified “compliant collapse” as the predominant mechanism of airway closure in initially patent airways during ARDS. Theoretical work suggests that this mechanism arises from instabilities in the airway liquid lining layer: local thickening of the fluid film generates asymmetric compressive forces that cause the airway wall to buckle, leading to lumen occlusion along a segment of its length. Analysis of airway cross-sections along their longitudinal axis revealed that collapse can occur at multiple sites within the same airway, sometimes resulting in gas trapping and local overdistension. The involvement of fluid-layer dynamics at microscopic scales indicates that closure and reopening depend not only on critical pressures, but also on time-dependent processes such as fluid redistribution and wall buckling kinetics [51].

Taken together, the above findings support a picture of the acutely injured lung as a highly dynamic, mechanically heterogeneous system, in which ventilation patterns emerge from the interplay of regional elastance, viscoelastic time-dependence, airway–parenchymal interdependence, and microscale fluid mechanics. Cyclic R/D is intrinsically heterogeneous, even within clusters of acini exposed to identical macroscopic pressures. This heterogeneity originates at least from the conjunction of two properties: the time-dependent nature of R/D, and mechanical interdependence between adjacent units (Fig. 3B). Regions experiencing cyclic R/D exhibit greater inflammatory cell infiltration and molecular signaling, providing direct evidence for mechano-inflammatory coupling. Small-airway closure in ARDS is dominated by compliant collapse, a fluid–structure instability driven by increases in the airway liquid lining layer surface tension. Importantly, compliant collapse is also time-dependent, influenced by lining fluid redistribution and wall buckling kinetics, and may provide a mechanistic framework for the time-dependence of R/D (Fig. 3C). PEEP, although beneficial for gas exchange, cannot fully suppress R/D or airway closure, and may redistribute both ventilation and perfusion in ways that can promote dead space, pulmonary vascular resistance, hence right ventricular afterload [52].

#### Alveolar and microscale strain, VILI, and ECM remodeling

**Fig. 6:**
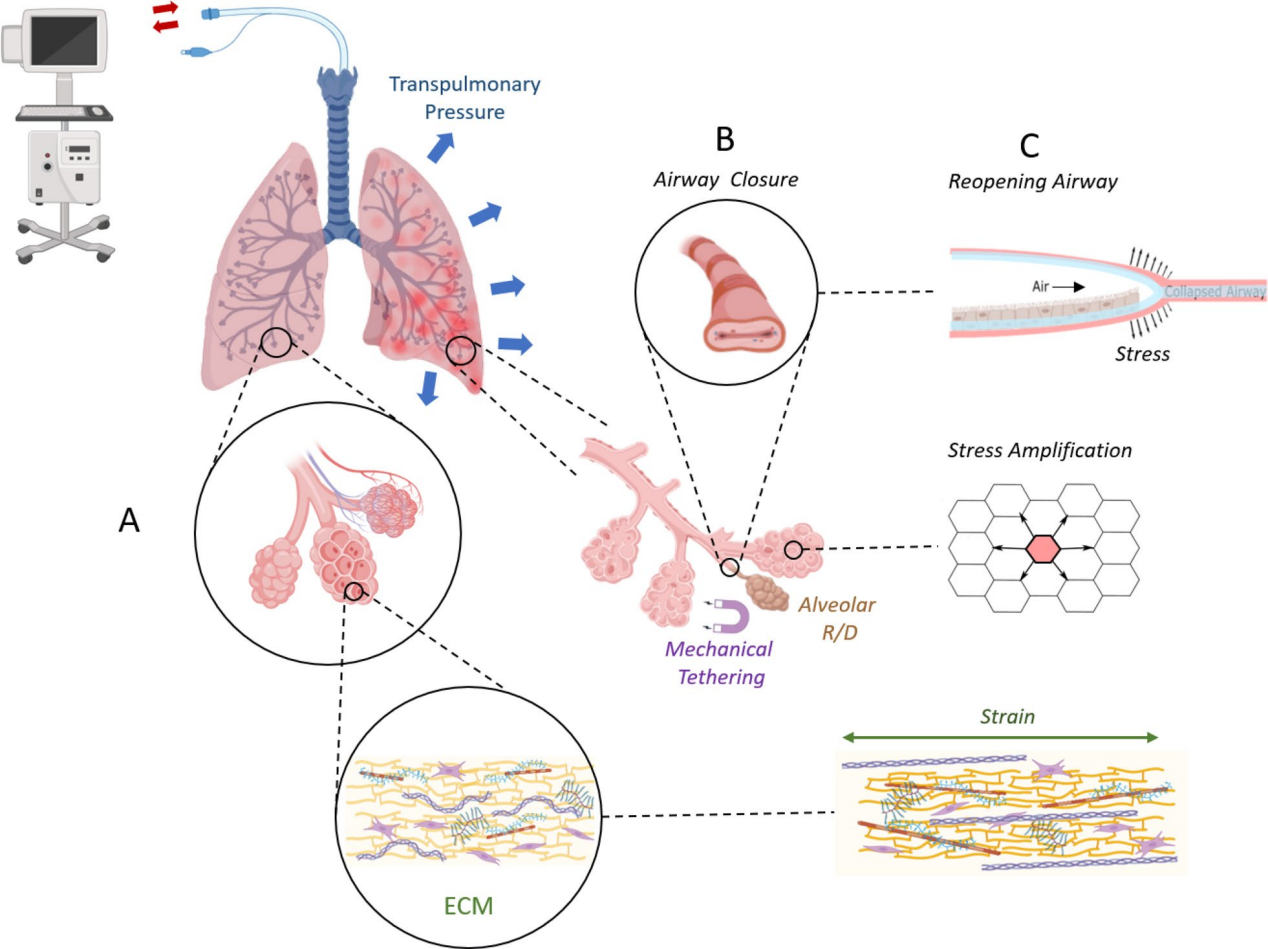
Multiscale organization of the lung. **A** Normal lung, acini, extracellular matrix (ECM) [94]; **B** injured lung; dynamics of alveolar recruitment/derecruitment (R/D), due to mechanical tethering between adjacent acinar structures [41], and ‘compliant collapse’ and reopening of small airways [51], due to cyclic changes in transpulmonary pressure during positive pressure ventilation; **C** reopening of closed airways causes abnormal fluid mechanical stresses along the airway walls [95]; open alveoli adjacent to closed ones undergo stress amplification which puts them at risk of injury extension [96]; strain within the extracellular matrix (ECM) elongates collagen fibers causing reorganization of fibril D-spacing [59]

The lung has an overdesigned architecture, with numerous components at multiple scales, from molecular to cellular, to tissue and whole organ, interacting together as a complex, dynamical system. The macroscopic mechanical behavior of the lung ‘emerges’ from these interactions, meaning that mechanical behavior at the organ level is not simply the sum, but rather an ensemble behavior of constituents at the microscale [31]. This multiscale organization is illustrated in Fig. 6.

The lung extracellular matrix (ECM) provides the cells with structural support, but also transmits external physical forces to cells [53]. Local strain can trigger downstream cellular signaling cascades through mechanotransduction that modifies cellular structure and function, involving numerous processes including cell contractility and myosin light chain phosphorylation, cell rheology, focal adhesion assembly, angiogenesis and ECM remodeling and homeostasis [53]. However, data on the local microscopic deformation of peripheral airspaces induced by mechanical ventilation are very limited, despite their importance for understanding mechanisms at the onset of ventilator-induced lung injury (VILI).

This is due to the technical difficulty of imaging deep lung tissue structures at microscopic resolution in vivo. The lung tissue has dynamic viscoelastic properties. Unlike elastic materials that deform instantaneously in response to stress loading, like many biological tissues, the response of the lung parenchyma to stress is delayed. It is impeded by internal frictional forces, and the rearrangement of internal components in order to dissipate applied forces, such as during positive pressure ventilation [54]. These frictional forces are determined by the constituents of the lung ECM including collagen and elastin and a ground substance composed of proteoglycans (PGs) and glycosaminoglycans (GAGs) [55]. The viscoelastic properties of the lung tissue are also influenced by adherent cells, the surface tension of the air–liquid interface and the organization of the parenchyma [53]. In the normal, homeostatic lung, mechanical behavior at low lung volumes is governed predominantly by surface tension forces generated by the surfactant layer lining the alveolar air–liquid interface. At higher lung volumes, however, the extracellular matrix becomes progressively engaged, and its structural components increasingly determine tissue mechanics and overall lung stability [53, 55].

The relationship between the strain-induced changes in molecular organization of the main fibrillar constituents of the ECM, the collagen and elastin fibers, and the in vivo lung tissue mechanical properties is not well known. Such a mechanistic link is valuable to better understand the pathophysiology of VILI, but also of other lung diseases affecting the ECM such as emphysema and fibrosis [53].

Using SR tomographic microscopy of precision-cut rat lung slices, Rausch and colleagues segmented volumetric images to provide a 3D alveolar geometry [56]. They used finite-element analysis to compute 3D strain distributions within the alveolar wall under controlled global deformation. They found that local principal strains could be several times higher than the applied macroscopic strain and that thin walls and junctions between neighboring alveoli were particularly prone to high strain. These results provide quantitative support for the idea that microscopic stress raisers may initiate VILI even when global tidal volumes are within protective ranges.

**Fig. 7:**
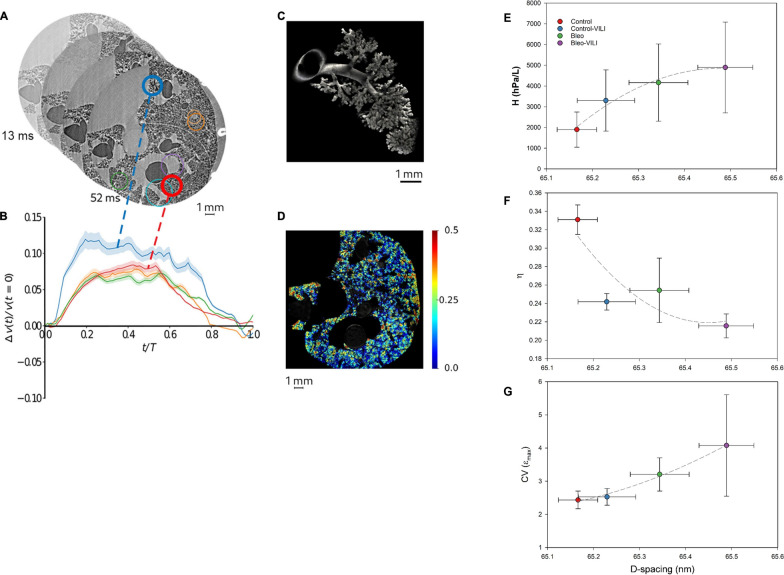
Quantitative mapping of lung tissue biomechanics in a live rat. **A** Sample sequential X-ray phase-contrast CT images at successive time points, reconstructed by retrospectively sorting of 250,000 individual 2 ms image projections with respect to the phase of heart contraction and breathing, yielding 78 time points during the breath; **B** regional strain as a function of time computed within airspaces in the regions of interest of same color as in panel (A). The shaded area represents within-ROI standard deviation; **C** a segmented airway with subtending conducting airways and terminal acinar structures at end-expiration in a live rat; **D** sample regional strain map of airspaces in vivo in the same animal. Color bars indicate strain (δV/Vt0, where t0 is the start of the breath). Reproduced from [57]. **E** Relation between collagen fiber D-spacing and tissue elastance (H); **F** relation between collagen fiber D-spacing and tissue hysteresis (η); **G** relation between collagen fiber D-spacing and local strain heterogeneity at maximum intra-breath lung expansion (CV(εmax)) [59]. Note that D-spacing increases with tissue stiffness, conversely, tissue hysteresis is reduced with D-spacing. Increased heterogeneity of local εmax is associated with increased collagen D-spacing. Control-VILI: normal lung exposed to high tidal volume (VT) ventilation; Bleo: bleomycin-injured lungs at 7 days; Bleo-VILI: bleomycin-injured lungs at 7 days subsequently exposed to high VT ventilation

Cercos et al. developed a 4D SR PC-CT at 6 μm^3^ voxel resolution and a higher temporal resolution than previously achieved (10 ms), in live anesthetized rats under controlled mechanical ventilation [57]. Using stepwise image-registration algorithms, they were able to compute maps of strain distribution due to positive-pressure breaths in the lung acini and in blood vessels (Fig. 7, A–D). In medium-sized blood vessels, the strain profiles resembled that of pulmonary arterial pressure profiles. Acinar deformation, on the other hand, was anisotropic, and dominated by alveolar expansion rather than ductal expansion or recruitment. This finding was later confirmed by higher resolution (0.67 µm^3^ voxel) in vivo SR microtomography in normal mouse lungs, although only in the very apical alveoli that are less subject to cardiovascular motion artifact [58].

Subsequently, Deyhle et al. [59] examined the impact of high tidal volume mechanical ventilation on the stiffness of local lung tissue utilizing 4D synchrotron phase-contrast micro-CT in both normal lungs and those subjected to bleomycin-induced injury seven days prior in anesthetized rats. Quantitative maps of local lung strain were generated within the aerated lung acini. At the nanoscale, the organization of collagen and elastin fibrils was assessed using SR SAXS. In the aerated acini of normal lungs following injurious ventilation, as well as in bleomycin-injured lungs, there was a reduction in local microscopic tissue deformation, which correlated with an increase in dynamic elastance as measured by oscillometry. The SAXS analysis revealed an increase in collagen fibril D-spacing due to injurious ventilation, suggesting elongation of collagen fibrils in both normal and bleomycin-injured lungs. A positive correlation was found between collagen periodicity and global tissue elastance, while an inverse correlation was observed with tissue hysteresis (Fig. 7, E–G). No such correlation was observed for elastin. These findings highlighted the influence of both inflammatory lung injury and high-strain mechanical ventilation on the nanoscale fibrillar structure of collagen, and for the first time, established a link between collagen D-spacing and the stiffening and viscoelastic behavior of global lung tissue. The current model for interpreting these results suggests that small strains lead to the unfolding of crimps along collagen fibrils in their relaxed state [60]. As strain increases, thermally induced molecular kinks, which are believed to occur within the gap region (Fig. 1F), become straightened, resulting in fibril elongation. Additionally, lateral ordering increases, causing fibril orientation to shift toward the direction of the strain [61, 62]. Beyond this point, further strain increases result in stretching of the triple helices and cross-links between them, permitting side-by-side sliding of adjacent collagen molecules, thereby enhancing D-spacing.

Together, these data confirm the multiscale nature of lung mechanics in the injured lung, and highlight the role of SR micro-CT imaging as a valuable tool in exploring it.

### Regional lung function in acute airway obstruction

Acute respiratory failure can also result from severe airway obstruction. In preclinical models, bronchoconstriction induced by the inhalation of histamine [63] or methacholine [52] produces patchy ventilation defects interspersed with zones of increased ventilation, consistent with self-organized pattern formation predicted by computational models of airway–parenchymal interaction [64]. The kinetics of airway response to histamine can be very different in central versus small peripheral airways, with faster constriction and recovery in the lung periphery. KES-CT imaging studies showed that central airways were slower to reach their maximal constriction, occasionally showing paradoxical dilatations [65]. This phenomenon may be due to the dynamic tidal expansion of proximal bronchi when distal airways constrict downstream, which has been termed *serial* airway interdependence [66]. The pathway through which airway smooth muscle constriction is induced results in radically different patterns of airway constriction and ventilation distribution [67]. In rabbits sensitized to ovalbumin, airway constriction was induced either non-specifically through a pharmacological agent such as methacholine, which acts directly on the airway smooth muscle, or specifically through an allergen such as ovalbumin following allergic sensitization, which constricts the airways indirectly. While methacholine induced mainly central airway constriction, the allergen caused a much more heterogeneous pattern of ventilation distribution in the lung periphery through constriction of peripheral airways. Global responses in airway resistance and tissue elastance measured by oscillometry were demonstrated to be related to the central and peripheral airway responses, respectively [67]. Since both constricting agents were administered intravenously, the more inhomogeneous airway reaction in allergic animals may have been due to the local heterogeneity of the distribution of the immune cells involved in the allergic reaction itself. This was investigated using KES-CT imaging in ovalbumin-sensitized Brown-Norway rats, a species genetically prone to develop allergic sensitization [68]. When the animals were exposed to an allergen, and subsequently imaged with stable xenon, patchy regions of ventilation defects developed in the lung periphery. Histologic examination of matching slices of the lung tissue revealed that total cellular, eosinophil and mast cell infiltrations were stronger within, compared to outside the lung regions with ventilation defects. Moreover, the inhomogeneous distribution of an inhaled aerosol containing environmental antigens or constricting agonists can result in an inhomogeneous peripheral airway response. The regional deposition of aerosol particles containing iodine was quantitatively mapped in rabbit lungs [69]. This study showed that the deposition of aerosol particles of a median aerodynamic diameter of ~ 3μm was largely inhomogeneous, and was quantitatively reduced following methacholine-induced airway constriction.

In a model of ARF during severe histamine-induced bronchoconstriction, Porra et al. used KES-CT to show that PEEP can have beneficial effects on regional ventilation inhomogeneity [70]. Applying a moderate PEEP of 5 cmH_2_O increased the area of well-ventilated regions and reduced specific ventilation heterogeneity, at the cost of mild hyperinflation. These results suggest that PEEP can dilate flow-limited airways and recruit closed units when applied at moderate levels, decreasing the risk of critical airway closure.

**Fig. 8:**
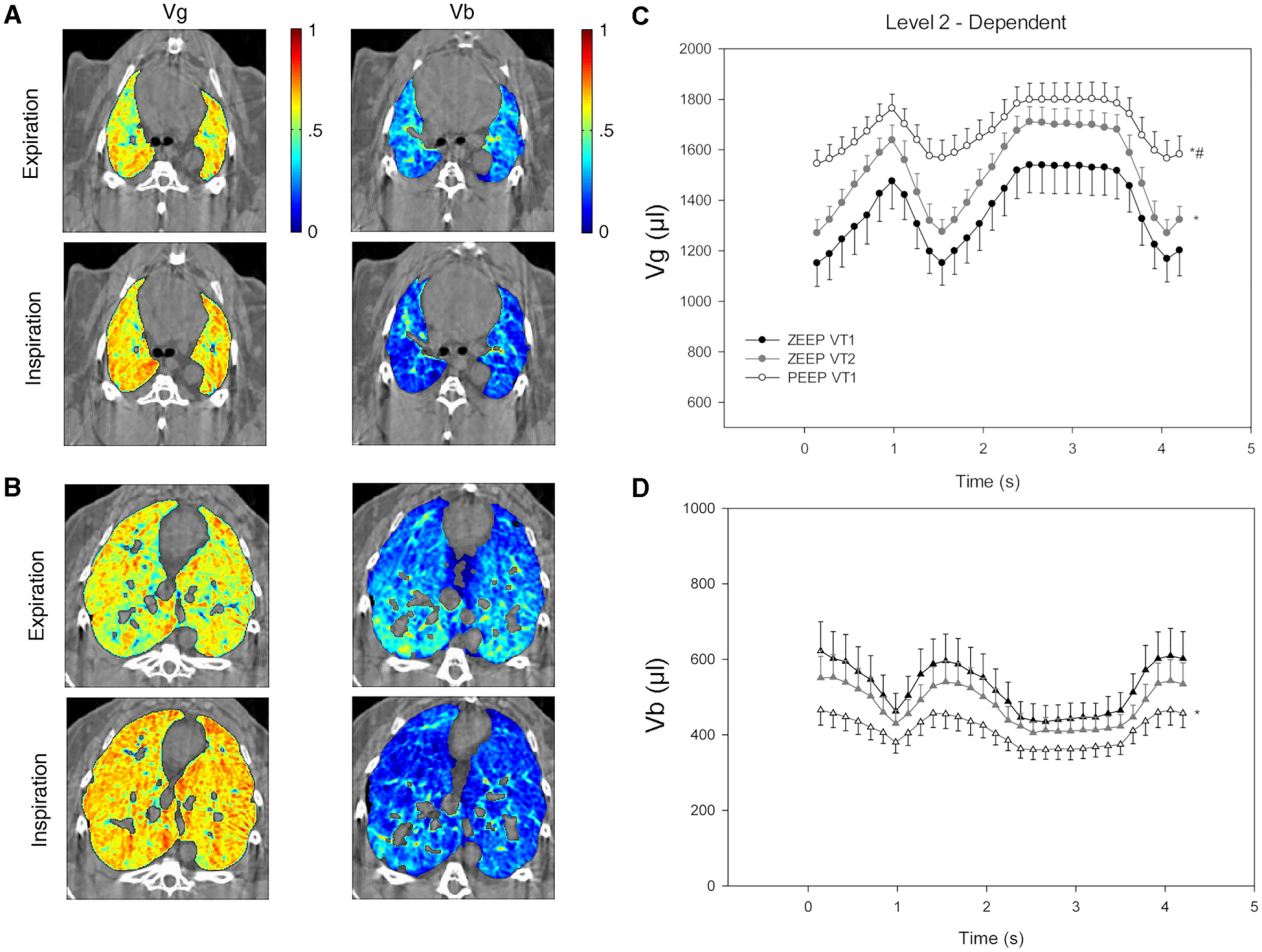
Representative composite synchrotron 4D K-edge subtraction CT images showing the regional distribution of fractional gas (gas volume [Vg]; left column) and blood (blood volume [Vb]; right column) within nondependent (**A**) and dependent (**B**) lung regions, at end-expiration (top rows) and end-inspiration (bottom rows), in a representative animal at zero positive end-expiratory pressure (PEEP). Graphs show the time evolution of the mean regional lung Vg (**C**) and Vb (**D**) in the dependent mage slices during two successive respiratory cycles with and without an end-inspiratory pause, at different tidal volume (VT) and PEEP combinations. Data are mean (± SE) (*n* = 6). *P < 0.05 versus VT1, zero end-expiratory pressure (ZEEP); #*P* < 0.05 versus VT2, 0 PEEP. Corresponding video animations showing the dynamic changes within the respiratory cycle are included in [52]. Reproduced with permission from [52]

However, improving the ventilation homogeneity in itself does not directly address gas exchange efficiency, which must depend on the coexisting distribution of perfusion [71, 72]. Indeed, 4D KES-CT has enabled the assessment of the dynamic within-breath changes of regional lung gas and blood volume in rabbits under positive pressure ventilation, and the effect of gravity, tidal volume (VT), PEEP, and inspiratory to expiratory (I:E) time ratio [52] (Fig. 8). They showed that regional lung perfused blood volume (PBV) cyclically decreases with lung inflation at each breath, with a larger magnitude of PBV swings in the dependent lung. Although an increase in VT did not have a significant effect, a modest PEEP of 9 cmH_2_O significantly decreased PBV. Also, increasing I:E through an end-inspiratory pause causes further dynamic drops in PBV. These data suggest that by reducing PBV in normally aerated lung regions, PEEP can promote dead space, possibly through different mechanisms including a reduction in systemic venous return and right ventricular preload [73], as well as an increased pulmonary vascular resistance and right ventricular afterload. The latter mechanism is due to the compression and elongation of intra-alveolar micro-vessels as alveolar pressure rises during positive pressure inspiration [74, 75]. These data had translational significance in that they helped design the appropriate ventilation pattern for capnodynamic measurements of end-expiratory lung volume (EELV) in ventilated patients [76]. The capnodynamic method induces cyclic changes in the alveolar concentration of CO_2_ through respiratory pauses [77]. The kinetics of expired CO_2_ measured by the ventilator following a pause are then used to compute EELV based on the Fick principle. Expiratory rather than inspiratory pauses were implemented based on the synchrotron 4D imaging studies [78], resulting in more accurate measurements, particularly when PEEP is applied.

KES-CT of both ventilation and PBV has provided further insight into the relation between ventilation–perfusion V_A_/$\dot{Q}$ mismatch and paradoxically worsening gas exchange after bronchodilator administration in acute bronchoconstriction models of respiratory failure. Recently, using KES-CT in a model of severe bronchoconstriction induced by methacholine in mechanically ventilated rabbit, Bayat et al. showed that despite a significant improvement in respiratory elastance by salbutamol, gas exchange worsened due to increased perfusion of low V_A_/Q̇ regions and increased shunt fraction [79]. Moreover, the phase III slope of exhaled CO_2_ was significantly related to V_A_/$\dot{Q}$ ratio and V_A_/$\dot{Q}$ inhomogeneity.

### Translational implications for ventilation strategies and clinical imaging

What do these mechanistic insights imply for the bedside management of patients with acute respiratory failure? First, they offer strong support for the principle that ‘one-size-fits-all’ ventilator settings are unlikely to be optimal in a mechanically heterogeneous lung in ARF. The emerging paradigm based on SR imaging studies is that mechanical ventilation settings should be personalized for optimal lung protection, and that assisted ventilation of ARF requires new and physiologically informed personalized protective ventilation strategies.

Second, SR imaging data highlight the importance of the dynamic, time-dependent, rather than purely static, mechanical behavior of the lung at the microscale. Recruitment–derecruitment and airway closure operate on time scales comparable to the respiratory cycle and are sensitive to the duration of low-pressure phases. Therefore, strategies that limit the time spent at low end-expiratory pressure, such as avoiding prolonged expiratory phases, may help mitigate airway collapse and its associated mechanical injury. The dynamic nature of R/D has also provided a theoretical basis for variable, rather than monotonous driving pressures [80–82]. Moreover, Broche et al. speculated that small amplitude flow oscillations due to R/D phenomena could be used to monitor R/D continuously in mechanically ventilated patients with ARDS and hence may allow personalizing mechanical ventilation settings aiming at minimizing R/D [41].

Third, these results call into question the exclusive reliance on airway pressures and tidal volume as surrogates of lung stress. Local strain amplification, stress raisers at interfaces, and occult airway closure all imply that patients with similar plateau pressures may experience very different micromechanical environments. Emerging bedside imaging techniques are needed for non-invasive online appreciation of parenchymal overdistension and R/D. Electrical impedance tomography appears as a promising candidate technique. It allows personalized PEEP selection [83], but also the assessment of ventilation and perfusion distribution at bedside as well as their changes with body position [84].

Finally, synchrotron imaging has an important role in the development and validation of computational models and digital twins of the lung [41, 85, 86]. High-resolution data on regional ventilation, strain, and airway behavior provide ground truth for model calibration and testing. Such models could ultimately be used to simulate patient-specific responses to different ventilatory settings and to optimize strategies that minimize VILI while ensuring adequate gas exchange. However, computation time remains a major bottleneck for digital twin-based approaches.

### Future directions

Although synchrotron facilities will remain scarce and specialized research platforms, their contributions to our understanding of VILI and ARDS are instrumental, mainly due to the capability of non-labeled microscopy, including in the intact in vivo lung. Future work will benefit from integrating synchrotron imaging with molecular and cellular readouts, such as spatial proteomics or multiplexed immunohistochemistry, to link local mechanical environments with specific inflammatory and remodeling pathways. Time-resolved studies over longer durations of ventilation and recovery will help to clarify how acute micromechanical events translate into global organ-level mechanics.

On the technological side, efforts are underway to transfer phase-contrast and K-edge concepts to compact X-ray sources and clinical scanners. Speckle-based and grating-based approaches compatible with the lower coherence radiation of compact sources may enable some of the benefits of synchrotron imaging at the laboratory or even clinical scale [87]. Phase-contrast imaging allows computing dark field image contrast, which is due to the local small angle scattering of X-rays by sub-resolution microscopic structures on the nanometer to hundreds-of-micrometers scale [88, 89] that are inaccessible from the attenuation or phase-contrast images [90]. The dark field imaging signal is decreased in bleomycin-injured lung [91, 92] and ventilator-induced lung injury [93], suggesting that this signal is related to the density of air–tissue interfaces, and may indirectly reflect alveolar size.

Machine learning-based reconstruction and segmentation methods, already being explored in the context of speckle-based CT [13], will be essential for handling the large data volumes and for extracting meaningful metrics.

## Conclusions

By revealing how ventilation, perfusion and strain are distributed in space and time, how small airways behave, and how components of the ECM are remodeled in models of ARF, synchrotron radiation imaging techniques provide new insight into the mechanisms of ventilator-induced lung injury and for designing more protective ventilation strategies. While synchrotron facilities themselves are not widely or clinically available, the concepts and insights they have generated are directly relevant to bedside practice and to the development of next-generation imaging modalities. Elucidating the 3D structure and real-time lung function at microscopic length scales in vivo is one of the most challenging applications of SR imaging. Continued interaction between SR imaging, computational modeling, and clinical research will be crucial to translate these insights into improved outcomes for patients with acute respiratory failure.

## Take home message

Synchrotron radiation imaging has advanced our understanding of lung function under mechanical ventilation in acute respiratory failure by revealing the dynamics of ventilation, perfusion, strain, small-airway behavior, and extracellular matrix remodeling at microscopic scales. The mechanistic insights gained from these techniques may inform protective ventilation strategies through close integration with computational modeling and clinical research.

## Data Availability

The data of the original manuscripts cited within this review are available through reasonable request from the corresponding author.
